# The Efficacy of the BioFire FilmArray Gastrointestinal Panel to Reduce Hospital Costs Associated With Contact Isolation: A Pragmatic Randomized Controlled Trial

**DOI:** 10.7759/cureus.27931

**Published:** 2022-08-12

**Authors:** Giulio DiDiodato, Ashley Allen, Nellie Bradbury, Julia Brown, Kelly Cruise, Christopher Jedrzejko, Valerie MacDonald, Jessica Pigeon, Amanda Sturgeon, Daniel Yellenik

**Affiliations:** 1 Critical Care Medicine, Royal Victoria Regional Health Centre, Barrie, CAN; 2 Infection Prevention and Control, Royal Victoria Regional Health Centre, Barrie, CAN; 3 Microbiology, Royal Victoria Regional Health Centre, Barrie, CAN; 4 Centre for Education and Research, Royal Victoria Regional Health Centre, Barrie, CAN

**Keywords:** infection prevention and control, randomized clinical trial, contact isolation, hospitalized patients, acute gastroenteritis, molecular testing

## Abstract

Background: Molecular syndromic panels can rapidly detect common pathogens responsible for acute gastroenteritis in hospitalized patients. Their impact on both patient and healthcare system outcomes is uncertain compared to conventional stool testing. This randomized trial evaluates the impact of molecular testing on in-hospital resource utilization compared to conventional stool testing.

Methods: Hospitalized patients with acute diarrheal illness were randomized 1:1 to either conventional or molecular stool testing with the BioFire FilmArray gastrointestinal panel (FGP). The primary outcome was the duration of contact isolation, and secondary outcomes included other in-hospital resource utilization such as diagnostic imaging and antimicrobial use.

Results: A total of 156 patients were randomized. Randomization resulted in a balanced allocation of patients across all three age strata (<18, 18-69, ≥70 years old). The proportion of positive stools was 20.5% vs 29.5% in the control and FGP groups, respectively (p=0.196). The median duration of contact isolation was 51 hours (interquartile range [iqr] 66) and 69 hours (iqr 81) in the conventional and FGP groups, respectively (p=0.0513). There were no significant differences in other in-hospital resource utilization between groups.

Conclusions: There were no differences in in-hospital resource utilization observed between the FGP and conventional stool testing groups.

## Introduction

Acute infectious gastroenteritis can be caused by viruses, bacteria, or parasites, resulting in a diarrheal illness that may be accompanied by fever, abdominal pain and/or cramping, hematochezia, nausea, and vomiting [1]. Between 12.5% and 25% of the population develop a gastrointestinal infection each year, with most cases being self-limiting and symptoms resolving within 14 days without treatment [2-3]. While the vast majority of the estimated 4 million Canadians who develop gastroenteritis have a mild and self-limited illness, approximately 9,250 to 14,150 are hospitalized each year with a mortality rate of between 1.6% and 2.2% [4].

Hospitalized patients with diarrheal disease will have stool samples collected and tested using standard microbiology methods that include culture for bacteria, nucleic acid amplification for viruses and bacteria, and microscopy or enzyme immunoassays for parasites [2]. The number of pathogens that can be identified is limited in most microbiology laboratories, and the turnaround time for reporting can take up to three days [2]. Recently, new nucleic acid amplification technologies have been developed that can test for multiple gastrointestinal pathogens in a single run, with results typically being reported in less than one day [5].

The BioFire® FilmArray gastrointestinal panel (FGP) is a multiplex polymerase chain reaction (PCR) test that can simultaneously test for 22 different viruses, bacteria, and parasites with excellent sensitivity and specificity with a one-hour turnaround time [6]. The FGP costs around $180 (Canadian 2020) (Nellie Bradbury, Microbiology Laboratory, Royal Victoria Regional Health Centre, Personal Communication), which is approximately 40% more expensive than conventional testing methods. Despite the test’s ability to detect more pathogens in a shorter period of time, a recent systematic review did not find any evidence to support a positive impact on either improved patient outcomes or cost-effectiveness compared to conventional testing [2].

To date, the available evidence on the cost-effectiveness of the FGP assay has been largely based on observational studies, usually historically controlled before-after designs associated with a high risk of bias [2,7-13]. The only study to be included in a systematic review of cost-effectiveness utilized a different PCR-based test, the Luminex xTAG [14], that has very similar diagnostic characteristics when compared to the FGP assay [15]. In the study by Goldenberg et al., differences in contact isolation days between conventional (observed) and PCR-based testing (simulated) were estimated and found to result in a 34.3% reduction in contact isolation days in the PCR-based group, a mean reduction of 0.94 contact isolation days per patient. Unfortunately, confidence intervals were not estimated for this point estimate. The cost of a single isolation day was reported as approximately £88 (United Kingdom) for fiscal 2011/2012. A breakeven analysis demonstrated that a reduction of 252 contact isolation days (11.4%) was needed to offset the increased costs associated with the Luminex xTAG assay. The cost of the Luminex xTAG assay was 0.4 times the cost of the FGP assay, suggesting that the number of contact isolation days needed to break even with the FGP assay could be as high as 635 days. In two recent conference reports about a randomised study in the United Kingdom that used the FGP assay in hospitalized patients with acute gastroenteritis, the authors estimated a significant reduction in contact isolation days in the FGP group compared to the conventional group [16-17]. This study was never registered, and a full report of the study has not yet appeared in a peer-reviewed journal, making it very difficult to evaluate the validity of the reported results.

There is uncertainty around both the clinical effectiveness and cost-effectiveness of the FGP assay compared to conventional testing for hospitalized patients with acute gastroenteritis. The aim of this study is to estimate the efficacy of the FGP assay to reduce contact isolation costs in hospitalized patients with acute gastroenteritis.

## Materials and methods

Study design

This was a single-centre, stratified (<18, 18 to 69, and 70 years of age with no fixed ratio), single-masked (patients), controlled, parallel, two-group, 1:1 allocation, pragmatic randomized trial conducted in Canada. This study followed the Consolidated Standards of Reporting Parallel Group Randomized Trials (CONSORT) guideline.

Study participants

Eligible participants were any patients admitted to a hospital with suspected acute gastroenteritis (AGE) in whom a physician requested a stool test(s) for viruses, bacteria, and/or parasites. Stool samples were collected at the earliest time after admission. This study was conducted under a waiver of consent due to minimal risk given that the FGP results have been shown to be concordant with conventional testing [18]. To avoid time delays from stool test reporting and decisions regarding contact isolation on the weekends or after 17:00 on weekdays that could confound the primary outcome, only participants whose stools were tested between Monday 08:00 and Friday 15:00 were eligible for inclusion. This was done to ensure that Infection Prevention and Control (IPAC) practitioners were available to review the stool testing results during their working hours (Monday 08:00 to Friday 16:00). In our hospital, IPAC practitioners are responsible for all decisions regarding additional precautions. Immunocompromised patients (HIV/AIDS, solid or stem cell transplant, febrile neutropenia, active chemotherapy, steroid treatment equivalent to prednisone ≥ 20 mg/day for ≥14 consecutive days preceding admission to hospital, active treatment with any of methotrexate, biologic immunosuppressants, or cyclophosphamide), nosocomial *Clostridioides difficile* infection (defined as a positive polymerase chain reaction test in any patient hospitalized ≥72 hours who develops diarrhea [≥3 loose bowel movements/day] in hospital), or any patient who develops diarrhea regardless of the length of hospital stay and has been hospitalized in the preceding three months for ≥48 hours, or who is being investigated as part of a possible diarrheal outbreak by either public health officials or IPAC were excluded from the study.

Study setting

The study took place at the Royal Victoria Regional Health Centre in Barrie, Ontario, Canada. The Royal Victoria Regional Health Centre is a 339-bed acute care, large community-teaching hospital. Barrie is a medium-sized city with a population of 150,000, located 100 kilometres north of Toronto in Central Ontario. Study recruitment started in December 2019. All consecutive patients admitted with acute gastroenteritis who met the eligibility requirements were enrolled in the study.

Study interventions

All stool samples were collected in an enteric pathogen transport (EPT) Cary-Blair medium and stored at a refrigeration temperature (2-8 °C) until processing. Eligible participants were randomly allocated to either stool testing with FGP or conventional methods by the microbiology laboratory technologist processing the stool. For the FGP group, 200 µL of stool was added to the BioFire® GI Panel pouch testing system. The pouch was inserted into the BioFire® FilmArray® instrument. This system can process one stool test every hour. For the conventional group, the microbiology laboratory performed all the following investigations. (1) Bacterial culture for Salmonella, Shigella, *Escherichia coli* O157 and Campylobacter - specimen in EPT medium planted to: (i) MacConkey agar, Sorbitol-MacConkey agar, Hektoen agar and Selenite broth (all from ThermoFisher) all incubated overnight at 35 °C; (ii) Campylobacter agar (ThermoFisher) incubated for 48 hours at 42 °C in a microaerophilic atmosphere. (2) Bacterial culture for Yersinia enterocolitica (≤ 18 years old): EPT specimen sent to Dynacare Laboratories (Brampton, ON, Canada) for processing, results back in 10-14 days. (3) Ova and parasites investigation: Sodium acetate-acetic acid-formalin specimen sent to the Public Health Laboratories (PHL) (https://www.publichealthontario.ca/en/laboratory-services/laboratory-contact) for testing, results back in 7-10 days

In the conventional stool testing group, separate physician orders would be required for the following assays: (1) Viral detection: rarely requested, requires a specimen in a sterile container, sent to the PHL for testing, results back in 5-7 days. The PHL assay is a real-time PCR for adenovirus, norovirus GI & GII, and rotavirus. (2) *C. difficile*: specimen in a sterile container, results in one hour (GeneXpert Intermedico, Cepheid, Sunnyvale, CA).

For some pathogens that are detectable in the FGP group, there may not be any conventional testing methods that permit isolation and identification (Sapovirus, Astrovirus, enteroaggregative *E. coli*, enteropathogenic *E. coli*, enterotoxigenic *E. coli*). For both groups, the microbiology laboratory technologists manually input all positive and negative stool testing results into the laboratory information system as soon as they become available, with an estimated delay of ≤30 minutes. The results were then automatically downloaded into the patient’s electronic medical record and the IPAC e-surveillance folder. IPAC can access all results as soon as they become available in their e-surveillance folder. The IPAC team used the Provincial Infectious Disease Advisory Council (PIDAC) best practice guidelines to direct the use of additional precautions during the study [19]. In general, all patients admitted to the hospital with acute diarrhea should receive additional precautions in addition to routine infection control practices. Additional precautions may include private room accommodation; contact precautions with personal protective equipment with gloves, masks, and gowns dependent on fecal incontinence; dedicated equipment; and additional cleaning measures as needed according to the etiologic pathogen. The decision to discontinue additional precautions is made by the infection prevention and control practitioners in our hospital and is dependent on the identification of a pathogen along with the control of diarrhea (continence and treatment where applicable). Where additional clarity was needed, the IPAC Medical Director was engaged. Physicians were not able to override any IPAC decisions regarding contact isolation precautions.

Study outcomes

The primary endpoint with respect to efficacy was the overall hospital costs associated with contact isolation. Differences in overall costs between groups were indirectly measured by assessing the differences in the duration of contact isolation precautions (hours). Secondary outcomes included differences in antimicrobial, diagnostic imaging, and endoscopy utilization during hospitalization. Antimicrobials included any antibacterials prescribed at the time of admission (empiric) or in response to a positive stool bacterial culture (directed), but did not include prescriptions for antifungals or antivirals. Diagnostic imaging includes any MRI, CT, or plain XR exam of the abdomen and/or pelvis. Endoscopic procedures include any sigmoidoscopy, colonoscopy, or oro-esophageal gastroduodenoscopy procedure. In addition, physicians’ perceptions of the value added by having the earlier results provided by the FGP assay were assessed using a two-item questionnaire. Physicians were asked to rank their agreement/disagreement with the following statements using a Likert scale (1=strongly disagree, 2=moderately disagree, 3=neither disagree nor agree, 4=moderately agree, 5=strongly agree): (1) without the earlier results provided by the FGP assay, my patient would have experienced possible/probable harm. (2) Because of the earlier results provided by the FGP assay, my patient’s treatment plan was changed (for example, change or discontinuation of antibiotic treatment, discontinuation of contact isolation, earlier discharge from hospital).

Sample size

The sample size that would be required to achieve an assurance of 0.8, or the unconditional probability that the trial would yield a statistically significant difference in the primary outcome, was estimated using a Bayesian approach [20]. The primary outcome difference was set at $140 (CDN) because this is equivalent to the net cost of the FGP assay at the Royal Victoria Regional Health Centre, the breakeven point for cost-neutrality. Using the estimated costs of a single contact isolation day of $100 (Canadian 2020) [21] and $135 (Canadian 2020) [14] from previous studies, a reduction of between 1.04 days (25 hours) and 1.4 days (33.6 hours) per patient would be needed to demonstrate cost-neutrality. Two previous studies estimated that the reduction in contact isolation days per patient were 94 [14] and 0.9 [16]. These two estimates of effect size were used as equally weighted prior information in our sample size estimates. Using a posterior standard deviation of $150 [14], the total sample size needed to detect an effect size of $140 (Canadian 2020) with an assurance of 0.8 and two-sided =0.05 ranged from 125 to 218 participants, assuming that 30% of the PCR-based assays detect *C. difficile* infection and that the probability of being placed in contact isolation for patients with a diarrheal illness was 50% [2,14]. Using the approach by Goldenberg et al. [14], the total sample size was estimated from the following equation = 2 (groups) * sample size per group * 1.3 (to account for the *C. ​​​​​difficile* infection rate of 30%) * 2 (for contact isolation rate of 50% of hospitalized patients with a diarrheal illness). It is unlikely that the posterior standard deviation would exceed the difference in contact isolation costs of $150 given that the observed range of contact isolation days in Goldenberg et al. [14] was from one to four days after excluding isolation due to *C.​​​​​​​ difficile*. nQuery 8 statistical software version 8.6.1.0 was used for all sample size calculations (GraphPad Software DBA Statistical Solutions, San Diego, CA).

Randomization and masking

A permuted block design was used to randomly allocate participants to either FGP or conventional testing in a 1:1 ratio within each stratum. Allocation was stratified by age (<18, 18 to 69, and ≥70 years of age). The block size was fixed at 4. There was no a priori fixed ratio for allocation in the different strata. The randomization table was created by an independent statistician using the ralloc command in STATA/MP 16.1 for Mac (StataCorp LLC, Texas, USA). The randomization table was uploaded into a secure, web-based software platform (REDCap®) hosted at the Royal Victoria Regional Health Centre [22]. The allocation sequence was concealed from researchers responsible for screening eligibility and enrollment. To enroll patients once they were deemed eligible, the researchers accessed the randomization module in REDCap® to assign patients to either FGP or conventional testing. Patients were masked from the assignment, but outcome assessors, healthcare providers, and analysts were not.

Statistical methods

Continuous variables were summarized using means and standard deviations (sd) or medians and interquartile ranges (iqr) depending on whether they were normally distributed or not. Frequency data were summarized using proportions. Comparisons of the frequency distribution of categorical data were done using Pearson’s chi-squared testing or Fisher’s exact testing. A comparison of means and proportions was done using t-tests and z-tests, respectively. A comparison of medians was done using the Wilcoxon rank-sum test. For the two-item questionnaire, physicians’ mean scores for questions 1 and 2 were compared to a hypothesized mean = 3 (=neither disagree nor agree). STATA/MP 16.1 for Mac (StataCorp LLC, Texas, USA) was used for all analyses. Statistical significance was defined a priori at p<0.05 (two-tailed) for all comparisons.

Research ethics approval

The study protocol and a waiver of informed consent were approved by the Royal Victoria Regional Health Centre Research Ethics Board on November 26, 2019 (R19-031). All methods were carried out in accordance with relevant guidelines and regulations.

This study has been published as a pre-print on Research Square (https://doi.org/10.21203/rs.3.rs-846035/v1).

## Results

**Figure 1:**
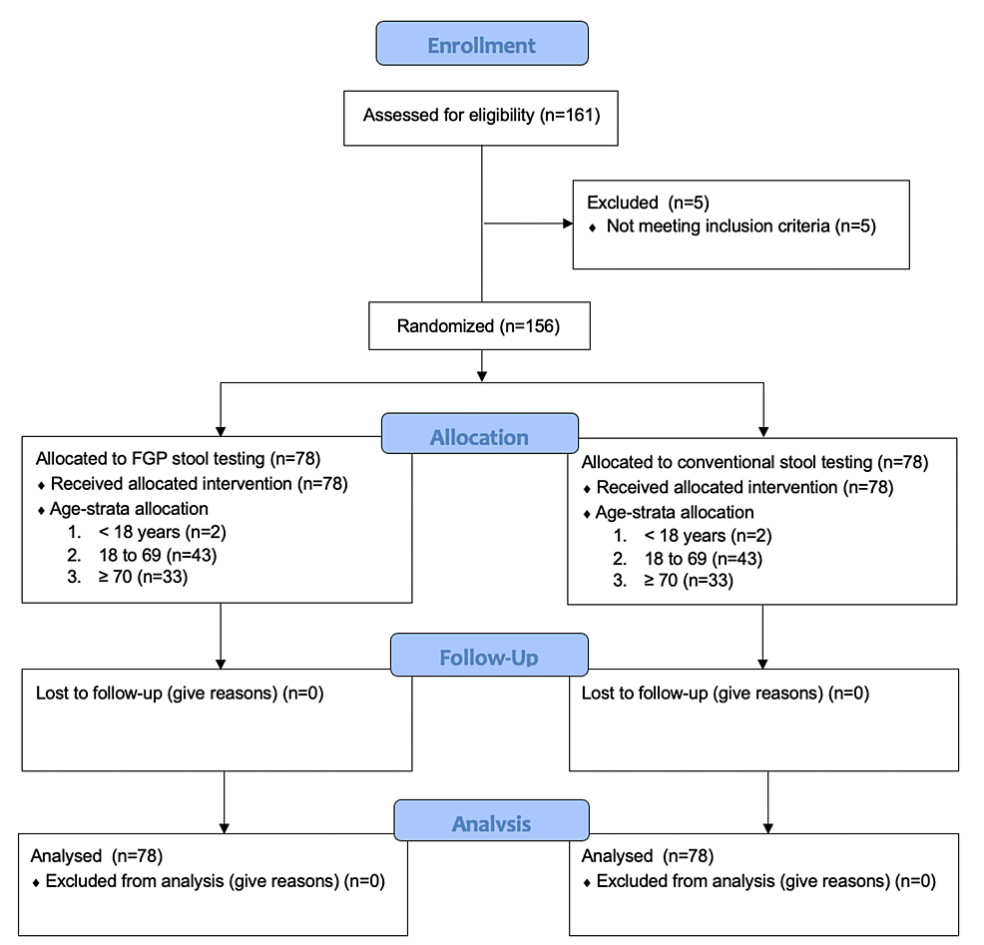
CONSORT patient flow diagram. CONSORT: Consolidated Standards of Reporting Parallel Group Randomized Trials.

A total of 156 participants were enrolled in the study from December 2019 to December 2020 (Figure 1).

The final sample size was less than planned as a result of slower than expected recruitment that was attributable to the COVID-19 pandemic. Both groups had complete follow-up for all patients, with 72 participants in each group being discharged from the hospital alive and the remaining 12 participants dying in the hospital. The groups were balanced across age strata, temporal recruitment pattern, and baseline characteristics (Table 1).

**Table 1:** Enrollment and baseline characteristics. ^1^Chi-squared(12)=5.5198, p=0.938 (Fisher’s exact p=0.945). ^2^Chi-squared(1)=0.6462, p=0.421. ^3^Chi-squared(4)=0.7022, p=0.951 (Fisher’s exact p=0.961); patients may have ≥1 bowel disease. ^4^Chi-squared(2)=1.333, p=0.513 (Fisher’s exact p=1.000). ^5^Chi-squared(3)=2.34, p=0.5105 (Fisher’s exact p=1.000). ^6^Chi-squared(1)=0.0298, p=0.863. ^7^Chi-squared(8)=4.1496, p=0.843 (Fisher’s exact p=0.844); patients may have ≥1 indication.

Variable	Conventional	FGP
Enrollment period^1^		
2019m12	2	1
2020m1	4	6
2020m2	5	5
2020m3	8	6
2020m4	2	2
2020m5	2	5
2020m6	9	4
2020m7	8	11
2020m8	6	6
2020m9	8	7
2020m10	7	10
2020m11	10	8
2020m12	7	7
Sex^2^		
Female	45 (57.7%)	40 (51.3%)
Bowel disease^3^		
None	56 (71.8%)	55 (70.5%)
Crohn’s	3 (3.9%)	3 (3.9%)
Ulcerative colitis	3 (3.9%)	5 (6.4%)
Celiac disease	0	0
Microscopic colitis	0	0
Irritable bowel syndrome	4 (5.1%)	4 (5.1%)
Other	14 (18.0%)	17 (21.8%)
Ostomy^4^		
None	76 (97.4%)	76 (97.4%)
Colostomy	1 (1.3%)	0
Ileostomy	1 (1.3%)	2 (2.6%)
Gastrointestinal surgery in the preceding 3 months^5^		
None	75 (96.2%)	76 (97.4%)
Cholecystectomy	1 (1.3%)	0
Pancreatectomy	0	0
Bowel resection	1 (1.3%)	0
Other	1 (1.3%)	2 (2.6%)
Antimicrobials in the preceding 3 months^6^		
Yes	25 (32.1%)	24 (30.8%)
Indications for testing^7^		
Fever	16 (20.5%)	10 (12.8%)
Abdominal pain	30 (38.5%)	35 (44.9%)
Bloody stool	14 (18.0%)	16 (20.5%)
Mucous in stool	0	0
Abnormal WBC	16 (20.5%)	24 (30.8%)
Hypotension	3 (3.9%)	2 (2.6%)
Tachycardia	2 (2.6%)	1 (1.3%)
Chronic diarrhea	28 (35.9%)	27 (34.6%)
Confusion	3 (3.9%)	3 (3.9%)
Elevated lactate	3 (3.9%)	2 (2.6%)

The percentage of stool assays that were positive was 20.5% (n=16) and 29.5% (n=23) in the conventional and FGP groups, respectively (2(1)=1.6752, p=0.196). There was no difference in the pattern of pathogens identified between the two groups (Table 2).

**Table 2:** Pathogens identified by conventional and FGP stool assays. ^1^Chi-squared(10)=10.2401, p=0.420 (Fisher’s exact p=0.506). FGP: FilmArray gastrointestinal panel, EPEC: enteropathogenic *E. coli, *ETEC: enterotoxigenic *E. coli*, STEC: Shiga-like toxin-producing *E. coli*.

Pathogen^1^	Conventional	FGP
Adenovirus	0	1 (4.4%)
Blastocystis hominis	2 (12.5%)	0
Clostridioides difficile	9 (56.3%)	15 (65.2%)
EPEC	1 (6.3%)	2 (8.7%)
ETEC	0	1 (4.4%)
Salmonella spp.	1 (6.3%)	2 (8.7%)
Sapovirus	1 (6.3%)	0
STEC	0	1 (4.4%)
Vibrio cholerae	0	1 (4.4%)
Vibrio non-cholerae	1 (6.3%)	0
Yersinia enterocolitica	1 (6.3%)	0
Total	16	23

There were no co-infections identified in either group. In-hospital resource allocation was similar between the two groups (Table 3).

**Table 3:** In-hospital resource utilization for the conventional and FGP groups. ^1^Chi-squared(2)=4.8889, p=0.087 (Fisher exact p=0.102). ^2^The reasons for never being placed in contact isolation were not collected. ^3^Chi-squared(1)=0.0258, p=0.872. ^4^Chi-squared(1)=2.8846, p=0.089. ^5^Wilcoxon rank-sum z=0.412, p=0.6802. ^6^Chi-squared(1)=1.1143, p=0.291; patients may have had ≥ 1 abdominal diagnostic imaging study. ^7^Chi-squared(2)=0.2956, p=0.863. ^8^Chi-squared(1)=0.1651, p=0.685.

Resource	Conventional	FGIP
Contact isolation^1^		
Prior to stool results	67 (85.9%)	67 (85.9%)
After stool results	0	4 (5.1%)
Never placed^2^	11 (14.1%)	7 (9.0%)
Terminal room cleaning^3^		
Yes	36 (46.2%)	35 (44.9%)
Antimicrobial treatment		
Yes^4^	47 (60.3%)	57 (73.1%)
Median duration (iqr) (hours)^5^	144 (216)	122 (144)
Changed in response to stool assay results	4 (8.5%)	7 (12.3%)
Diagnostic imaging		
Yes^6^	68 (87.2%)	72 (92.3%)
CT^7^	50 (73.5%)	56 (77.8%)
MRI	4 (5.9%)	6 (8.3%)
XR	43 (63.2%)	45 (62.5%)
Endoscopy		
Yes^8^	16 (20.5%)	14 (18.2%)
Sigmoidoscopy^9^	5 (31.3%)	5 (35.7%)
Colonoscopy	7 (43.8%)	7 (50.0%)
Oro-esophageal gastroduodenoscopy	4 (25.0%)	3 (21.4%)
Length of stay (days)		
Median (iqr)^10^	6 (13)	6 (11)

There was no difference in the duration of contact isolation between the two groups regardless of a positive or negative stool test (Table 4).

**Table 4:** Duration of contact isolation in conventional and FGP groups. ^1^Wilcoxon rank sum z=−1.949, p=0.0513. ^2^Wilcoxon rank sum z=−1.202, p=0.2293. ^3^Wilcoxon rank sum z=−1.190, p=0.2340. *** p<0.001 (Wilcoxon rank-sum test); ** p<0.01 (Wilcoxon rank-sum test); * p<0.05 (Wilcoxon rank-sum test).

Outcome (hours)	Conventional	FGIP
Duration of contact isolation		
Overall^1^		
Median (iqr)	51 (66)	69 (81)
Positive stool assay^2^		
Median (iqr)	80 (68)	123 (171)
Negative stool assay^2^		
Median (iqr)	45 (62)	59.5 (68.5)
Time to reporting of stool assay results		
Median (iqr)	48 (6)	3 (3)^***^
IPAC review (from the start of contact isolation)		
Median (iqr)	41 (66)	26 (30)^*^
IPAC review (from stool assay report)		
Median (iqr)	−23.5 (47)	0 (1)^***^
Time to discontinue contact isolation from IPAC review		
Median (iqr)	1 (51)	26 (91)^**^

Physicians (n=25, response rate=32.1%) had a mean score of 2.52 (standard deviation 1.29) and a mean score of 3.04 (standard deviation 1.49) on questions 1 (*my patient would have experienced harm without the FGP assay*) and 2 (*my patient’s treatment plan changed in response to the FGP assay’s earlier reporting results*), respectively. Neither of these scores differed from a hypothesized mean score of 3 (Q1 t-test=−1.8605, p=0.0751; Q2 t-test=0.1347, p=0.8940), where a score of 3 = neither agree nor disagree with the statement. 

## Discussion

Contrary to the only other randomized trial comparing FGP to conventional stool testing for hospitalized patients with suspected gastroenteritis [16], this study did not demonstrate any difference in the duration of contact isolation between these groups. In the study by Malachira et al. [16], the mean duration of contact isolation in the FGP group (n=70 participants) was 1.9 days (95% confidence interval 1.0 to 2.9) compared to 2.7 days (95% confidence interval 1.8 to 5.1) in the conventional group (n=70 participants) (p<0.001). This difference was reported to be due to differences between groups in which the stool assays were negative. In this study, the rate of positive assays did not differ between the two groups but was 44% and 23% in the FGP and conventional groups, respectively, in the Malachira et al. study. That study reported that 63% of FGP-negative patients had been correctly removed from isolation compared to only 28% in the conventional group (p=0.0012), although the criteria used for these decisions were never reported in either of the conference abstracts [16-17]. In this study, the decision to remove participants from contact isolation was made by IPAC practitioners using PIDAC guidelines [19]. These guidelines do not solely take into account results from diagnostic tests but are also dependent on symptom-based discontinuation criteria even in the presence of negative stool assays. This means that patients with negative FGP stool assays might still be kept in isolation if they did not demonstrate any improvement in their diarrheal illness, especially if they were incontinent or clinically worsening. This may help explain that despite the faster turnaround time of the FGP assay (median 3 hours [iqr 3]) compared to conventional testing (median 48 hours [iqr 6]) (p<0.001), and the insignificant lag between the reporting of the FGP stool assay results and their review by IPAC (median 0 hours [iqr 1]), the time required for IPAC to discontinue isolation in the FGP group was still over one day from the time of their FGP stool assay report review (median 26 hours [iqr 91]). This study also did not demonstrate any other differences in in-hospital resource utilization between these groups. In the study by Malachira et al., they commented on other outcomes of antibiotic use and length of stay but did not report the data. In an observational study by Axelrad et al. [7], small reductions in endoscopic procedures (8.4% [FGP] versus 9.6% [conventional] [p=0.008]) and abdominal radiographic imaging (29.4% [FGP] versus 31.7% [conventional] [p=002]) were reported among 9,402 patients who underwent FGP stool testing from March 2015 to May 2017 compared to 5,986 patients who underwent conventional testing from December 2012 to February 2015. In another pre-post observational study [8], a twofold reduction in abdominal radiographic imaging was seen between these groups (0.39 tests per patient [conventional] versus 0.18 [FGP] [p=0002]). In the observational study by Torres-Miranda et al. [13], the length of hospital stay was reduced by twofold in the FGP group (3.0 days) compared to the conventional group (7.5 days) (p=0.00002). All of these observational studies were at high risk of bias due to a lack of appropriate patient controls, selection bias, and temporal changes in clinical practices. A recent systematic review reported that there was an absence of evidence to support the cost-effectiveness of the FGP assay [2].

This study has several limitations. The definition of acute gastroenteritis was not specifically defined but was left to the discretion of the attending physician. This was a pragmatic decision meant to capture the real-world ordering practices of physicians in clinical practice. This lack of specificity in the definition could compromise both the exchangeability assumption and the stable unit treatment value assumption implicit in demonstrating causality in randomized trials. In addition, the number of stool assays directed against different pathogens ordered in the conventional arm might limit the beneficial impact of the FGP assay. However, the real value of the FGP assay compared to conventional testing is not necessarily the different number of pathogens that might be detected but the rapidity with which the results would become available, and so the number of assays ordered in the conventional arm should be less of a concern in limiting the study’s power to detect differences between the two groups in the primary outcome. The early termination of the study before recruiting the a priori sample size of 180 patients may have increased the risk of a type II error. However, when accounting for both a larger proportion of patients with *C. difficile* infection and those placed in contact isolation, the estimated sample size needed to demonstrate a statistically significant difference would have been reduced, making a type II error an unlikely explanation for the absence of an effect on the duration of contact isolation between the two groups. The trial was conducted during the COVID-19 pandemic, and this may have contributed to differences in additional precaution practices for all patients admitted to the hospital with diarrhea. However, the hospital employed a molecular test for COVID-19 testing with a turnaround time of one hour, suggesting that prolonged isolation would not have been due to testing delays. Other effects on IPAC practices from COVID-19 that may have contributed to prolonged contact isolation should have been equally distributed across both groups as a result of the random allocation schema. The criteria and processes for discontinuing contact isolation precautions used in this study may not be relevant to other organizations outside of Ontario, thus limiting the generalizability of the study results to other jurisdictions. Finally, the current price for the BioFire GI Panel has been reduced to $90 (Canadian 2020) (François Turgeon, Clinical Marketing Manage, bioMerieux, Personal Communication). While this lower price does not change the conclusions of this study, this lower price, its ease of use, and faster reporting times compared to conventional stool testing may have a significant impact on institutional decisions to supplant conventional testing with this assay.

## Conclusions

In this randomized trial, FGP stool testing did not result in any reductions in the duration of contact isolation compared to a control group of participants with acute gastroenteritis. Additionally, there were also no differences in antibiotic utilization, diagnostic imaging, endoscopic procedures, terminal room cleaning, or length of hospital stay between the two groups. Given these results, it is unclear if the FGP stool assay would reduce in-hospital resource utilization in a general hospital population admitted with gastroenteritis in the absence of concomitant policy changes that support earlier discontinuation of contact isolation precautions based on rapid diagnostic test results as opposed to historical criteria based on signs and symptoms of disease and culture-based test results.
